# Age-Related Incidence and Family History in Frontotemporal Dementia: Data from the Swedish Dementia Registry

**DOI:** 10.1371/journal.pone.0094901

**Published:** 2014-04-10

**Authors:** Christer Nilsson, Maria Landqvist Waldö, Karin Nilsson, Alexander Santillo, Susanna Vestberg

**Affiliations:** 1 Department of Memory Disorders, Skåne University Hospital, Lund, Sweden; 2 Clinical Memory Research Unit, Department of Clinical Sciences, Lund University, Lund, Sweden; 3 Geriatric Psychiatry Unit, Department of Clinical Sciences, Lund University, Lund, Sweden; 4 Department of Psychology, Lund University, Lund, Sweden; University of Manchester, United Kingdom

## Abstract

**Objectives:**

Frontotemporal dementia (FTD) is considered to be a mainly early-onset neurodegenerative disorder with a strong hereditary component. The aim of the study was to investigate age-related incidence and family history in FTD compared to other dementia disorders, especially Alzheimer's disease (AD).

**Methods:**

The Swedish Dementia Registry (SveDem) registers all new cases of dementia diagnosed by the participating centres, including data on demographics, diagnosis, and investigations used. Data for the period 2008–2011 were extracted and compared with age-related population data on a regional and national level.

**Results:**

There were 20 305 patients registered in SveDem during 2008–2011, whereof 352 received a diagnosis of FTD. Mean age at diagnosis for FTD was 69.6 years and almost 70% of FTD cases were 65 years or older at the time of diagnosis. Both FTD and AD showed an increased incidence with age, which reached a maximum in the age group 80–84 years at 6.04 and 202 cases per 100 000 person-years, respectively. The proportion of cases with a positive family history was significantly lower in FTD than in AD.

**Conclusions:**

Contrary to general opinion within the field, data from SveDem show that the incidence of FTD increases with age, and that the majority of cases are diagnosed after the age of 65 years. In addition, data from SveDem might suggest that the importance of hereditary factors in general is similar in FTD and AD. The recognition of these findings has important consequences for the diagnosis, treatment and care of patients with FTD.

## Introduction

The frontotemporal dementias (FTD) are a group of neurodegenerative disorders affecting primarily the frontal and temporal lobes of the brain, leading to various combinations of behavioural, cognitive and motor symptoms [1], [2]. Clinical syndromes within the FTD-spectrum include behavioural variant FTD (bvFTD), semantic dementia (SD), progressive non-fluent aphasia (PNFA) and FTD with motor neuron disease (FTD-MND) [1]. The underlying pathology is heterogeneous, with classification based on the main constituents of neuronal inclusions found at autopsy [3].

Although the disorder was first described by Pick more than 100 years ago, formal clinical and neuropathological criteria were not developed until the mid-90s [4], [5]. Accordingly, diagnosis and collection of demographic data on FTD have largely been performed in specialized centres with a research interest in the disorder [6]. Early case series and prevalence studies suggested that FTD is an early-onset disorder with a mean age at onset around 53 years [1], [7] and a prevalence of approximately 15–22 cases per 100 000 in the age group 45–64 years [2], [7]–[10], although lower figures have also been reported [11]. Studies of the incidence in FTD are much fewer, but have found similar rates in annual incidence for early-onset FTD (<65 years) of 2.7–4.1 cases per 100 000 person-years in the 45–64 year age group [9], [10], [12]–[14]. Although the existence of late-onset cases of FTD (≥65 years) has been recognized, with a reported range for age at onset of 21–84 years [1], [2], [6], [9], the view of FTD as an early-onset dementia has largely prevailed [1], [9], [10], [15]. However, more recent community-based studies have shown much higher incidence in late-onset FTD at 16.7 per 100 000 person-years, compared to 1.3 in early-onset FTD [14], [16]. It is thus possible that FTD might be underdiagnosed in the elderly population [2], [14], [16].

Many studies have reported that a high proportion of FTD cases have a positive family history for dementia [17]–[22]. Several known genetic mutations have been described that can lead to autosomal dominant hereditary FTD, estimated to account for approximately 10–30% of cases [2], [19]–[21]. The remainder of patients with FTD are presumed to be of sporadic origin, although it should be noted that no recessive mutations causing FTD have been described so far. On the basis of available evidence, it has been concluded that the proportion of familial cases is higher in FTD than in other major neurodegenerative disorders [1], [9]. However, the high community prevalence of other types of dementia in the elderly might make estimates of the proportion of familial cases uncertain in FTD [2].

The aim of the present study was to test the hypothesis that the incidence of FTD increases with age and is higher in the elderly, similarly to other neurodegenerative disorders. Furthermore, the proportion of FTD cases with a positive family history was compared to family history in AD. For this purpose we used data extracted from the Swedish Dementia Registry (SveDem) for the period 2008–2011, encompassing 20 305 new cases diagnosed with dementia, including 352 cases of FTD. The present study supports the hypothesis of increasing incidence of FTD with age, similar to other major neurodegenerative disorders, and that late-onset FTD is more common than early-onset FTD.

## Methods

### Study design

The Swedish Dementia Registry (SveDem; www.svedem.se), is a national quality registry on dementia disorders, financed by the Swedish Association of Local Authorities and Regions and the Swedish Brain Power network. Uppsala Clinical Research Centre is responsible for the development and support of the database online. SveDem was started in 2007 with the purpose of improving time to diagnosis, diagnostic workup, treatment and care of persons with dementia. New cases of dementia diagnosed at the participating primary care and specialist centres are registered. Almost all centres (95%) specializing in the diagnosis of dementia (i.e. memory clinics) in Sweden use the registry [23]. For the purpose of the present study, anonymized data on diagnosis, date of diagnosis, investigations used, age, gender, diagnosing centre, place of habitation and family history for individuals registered in SveDem during the time period 2008–2011 was extracted.

Diagnosis is registered in SveDem by choosing from a list of diagnoses (early-onset AD, late-onset AD, vascular dementia (VaD), Mixed AD and VaD, FTD, Dementia with Lewy bodies (DLB), Parkinson disease dementia (PDD), Dementia of other causes and Dementia not specified). In addition, the primary and secondary ICD-10 codes are registered. In Sweden, the ICD-10 codes used for the diagnosis of different forms of dementia are made according to a consensus document [24]. To avoid cases with mistaken registration or frontotemporal syndromes of other causes, only patients registered as frontotemporal dementia and the ICD-10 codes F02.0 plus G31.0 were considered as FTD with a neurodegenerative cause. Registration of diagnosis in SveDem does not differentiate between the different clinical syndromes of FTD (i.e. bvFTD, SD, PNFA or FTD-MND).

It is important here to explain the nature of the Swedish health care quality registries, such as SveDem. Data are entered in a standardized fashion to enable follow up of diagnostic procedures, treatment and management, with the purpose of standardizing and improving health care nationally. Extracted data for scientific purposes are anonymised and cannot be combined with the individual patient's medical records. Adherence to the former [4] and new [25] diagnostic criteria for FTD cannot therefore be controlled for registered cases. To improve diagnostic specificity, we therefore also analysed age data in a subgroup of patients including only cases diagnosed in specialist centres that had performed both cognitive testing and neuroimaging. In a third group, the data from cases that had performed lumbar puncture was analysed. Analysis of AD biomarkers (total-tau, phospho-tau and β-amyloid_1-42_) in cerebrospinal fluid (CSF) is routinely used in clinical practice in Sweden as part of the diagnostic procedure in the diagnosis of dementia and is always included if lumbar puncture is performed.

The presence or absence of a family history of dementia in first- and second-degree relatives is noted in the SveDem registration form. For classification purposes, the responses were divided into three categories: Positive family history (Yes), negative family history (No/None known) and Not known (reserved for when questions about family history have not been asked alternatively no response registered).

### Statistical analysis

Incidence rates were calculated by dividing the number of diagnosed cases with the number of person-years in the population at risk, multiplied by 100 000. This was done for each age cohort as well as for the whole population in defined geographical regions. Data on population size in each age group and in each local council was obtained from publicly available census figures for 2011 (Statistics Sweden; www.scb.se). The population at risk was calculated by subtracting the number of cases with established dementia in each age cohort using published estimates of dementia prevalence [14], [26].

Differences in gender distribution for each diagnosis, as well as the distribution between early-onset and late-onset cases of FTD, were calculated by an exact binomial test. T-test was used for statistical comparisons of age at diagnosis in AD and FTD. For comparison of the proportion of a positive family history in first and second-degree relatives between cases with AD and FTD we used Fisher's exact test. In cases with FTD, the proportion of cases with a positive family history in different age groups was compared using Fisher's exact test and Mantel-Haenszel chi-square.

The statistical analyses were performed in R version 2.15.2 (The R Foundation for Statistical Computing, http://www.r-project.org/) and SPSS Statistics 18 for Windows (IBM Corporation, Somers, NY, USA). A p-value below 0.05 was considered statistically significant.

### Ethics statement

The project was approved by the Regional Ethics Committee of Lund University (Permit number 2012/137) and the SveDem Steering Committee. All registered individuals were informed about SveDem orally and in writing at the time of diagnosis and gave their consent to registration, with the possibility of declining participation, in accordance with Swedish legislation.

## Results

In total, 20 305 cases of dementia were registered in SveDem during the period 2008–2011. The number of cases and proportion of the different diagnoses registered are summarized in Table 1. As expected, AD was the most common diagnosis and accounted for 53% of all cases, including cases with mixed AD and VaD. The range for age at diagnosis was very similar for all the diagnoses and the previously known gender differences in AD (female > male) and DLB/PDD (male > female) were confirmed (Table 1). In the FTD group there was a slightly higher proportion of females at 56% (95% CI 50.0–60.6), with borderline statistical significance (p = 0.049).

**Table 1:** Diagnosis, age at diagnosis and gender distribution in SveDem 2008-11.

Diagnosis	Cases (%)	Age (range)	F/M (p-value)
AD	6766 (33.3)	77.4 (27–99)	65.8/34.2 (p<0.0001)
AD+VaD	4064 (20.0)	80.9 (52–100)	59.3/40.7 (p<0.0001)
VaD	3652 (18.0)	79.8 (33–103)	50.5/49.5 (p = 0.53)
FTD	352 (1.7)	69.6 (39–96)	55.4/44.6 (p = 0.049)
DLB	477 (2.3)	76.8 (53–94)	38.2/61.8 (p<0.0001)
PDD	316 (1.6)	75.0 (49–94)	38.6/61.4 (p<0.0001)
Other	500 (2.5)	74.4 (30–95)	52.5/47.5 (p = 0.30)
Dementia NS	4178 (20.6)	80.7 (40–101)	63.2/36.8 (p<0.0001)
Total	20305 (100)	79.0 (27–103)	59.6/40.4 (p<0.0001)

Data are n (%) for diagnoses, mean age (range) and the female (F)/male (M) distribution percentage (p-value). Statistical differences in gender distribution was calculated by a binomial test, see Methods. AD  =  Alzheimer's disease, VaD  =  Vascular dementia, FTD  =  Frontotemporal dementia, DLB  =  Dementia with Lewy bodies, PDD  =  Parkinson disease dementia, Other  =  Dementia of other causes, Dementia NS  =  Dementia not specified.

### Diagnosis of FTD

There were 352 cases of FTD registered during the investigated time period. Diagnoses of FTD were made almost exclusively in specialist clinics (95%). The majority of centres that reported cases of FTD registered less than 6 cases (range 1–43). More than one third (37%) of the cases were registered in only 5 centres. While FTD accounted for 1.7% of all cases overall, the proportion was greater in centres with a higher number of FTD cases. For example, in our own centre (Lund) the proportion of FTD was 4.2%.

In Sweden, certain basic diagnostic procedures are considered mandatory for a diagnosis of dementia and have to be performed before referral to a specialist centre. These basic procedures include history from patient and caregiver, physical examination, blood chemistry, cognitive screening tests and computer tomography (CT) of the brain. Among all recorded FTD cases, 95.7% had performed cognitive testing of some kind (MMSE, other screening tests, and/or examination by a neuropsychologist). The main reason given for not performing cognitive tests was that it was not possible due to the patient's condition. Structural neuroimaging (CT or MRI) was performed in 96.3% and lumbar puncture in 64.4% of cases with FTD.

### Incidence of FTD

For calculation of the total incidence of FTD, we chose to compare the smaller regions of Lund and Uppsala, with populations of 289 550 and 335 000 inhabitants, respectively, with the larger Stockholm region with 2 091 357 inhabitants. While the Lund and Uppsala regions each are dominated by a specialist centre located at the respective university hospital, both with research on FTD, there are 10 non-university specialist centres in the Stockholm region in parallel to the memory clinic at Karolinska University Hospital. The total annual incidence of FTD in these three regions was 1.17 (Stockholm), 2.07 (Lund) and 3.21 (Uppsala) per 100 000 inhabitants.

**Figure 1:**
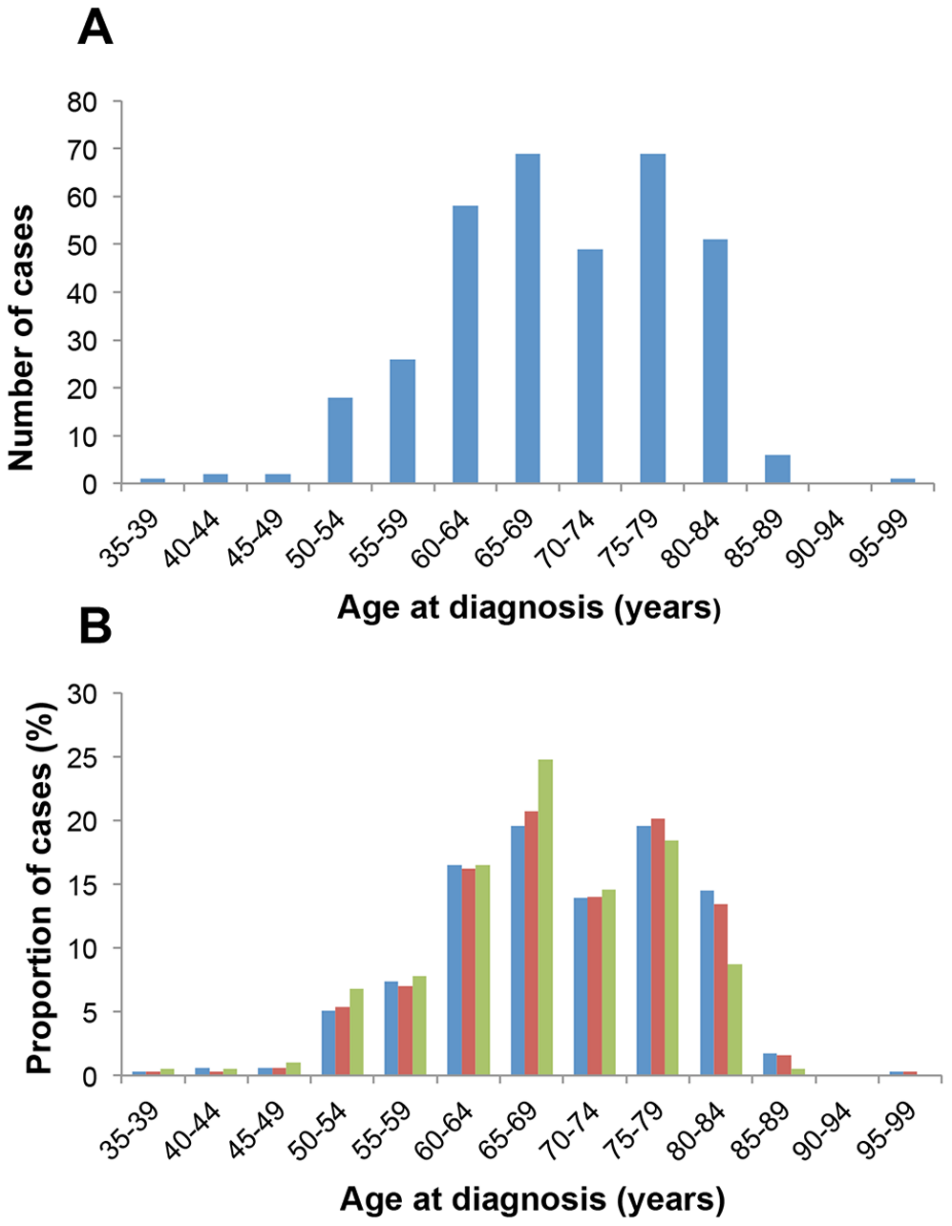
Age at diagnosis in FTD. (A) Age at diagnosis in FTD cases registered in SveDem 2008–2011. (B) The proportion of FTD cases in each age group in the total FTD cohort (blue bars), compared to those cases diagnosed in specialist centres where investigations included cognitive testing and structural or functional imaging (red bars) and cases that had performed lumbar puncture with CSF analysis (green bars).

The distribution of age at diagnosis in FTD cases is summarized in Fig 1A. There were significantly more FTD cases 65 years or older at the time of diagnosis (n = 245), than cases <65 years (n = 107). Early-onset cases represented 30.6% of total FTD cases (95% CI 25.2–35.0; p<0.0001).

Symptoms of frontal lobe dysfunction occur both in VaD and AD. To minimize the risk for incorrect diagnosis of FTD, especially in older age groups, we performed further analysis of the age distribution after excluding cases that did not fulfil the following criteria: (1) diagnosis at a specialized centre, (2) use of structural (CT/MRI) or functional (SPECT/PET) neuroimaging, (3) use of cognitive testing (MMSE, other screening tests, and/or examination by a neuropsychologist). Out of the 352 FTD cases, 314 cases fulfilled the above criteria. In addition, out of these 314 cases, the 206 cases that had performed lumbar puncture with CSF analysis of AD biomarkers were analysed separately. The age distribution in these three groups was very similar except for a slightly lower proportion of cases in the age group 80–84 years and a higher proportion of cases in the age group 65–69 years among cases that had performed lumbar puncture (Fig 1B).

**Figure 2:**
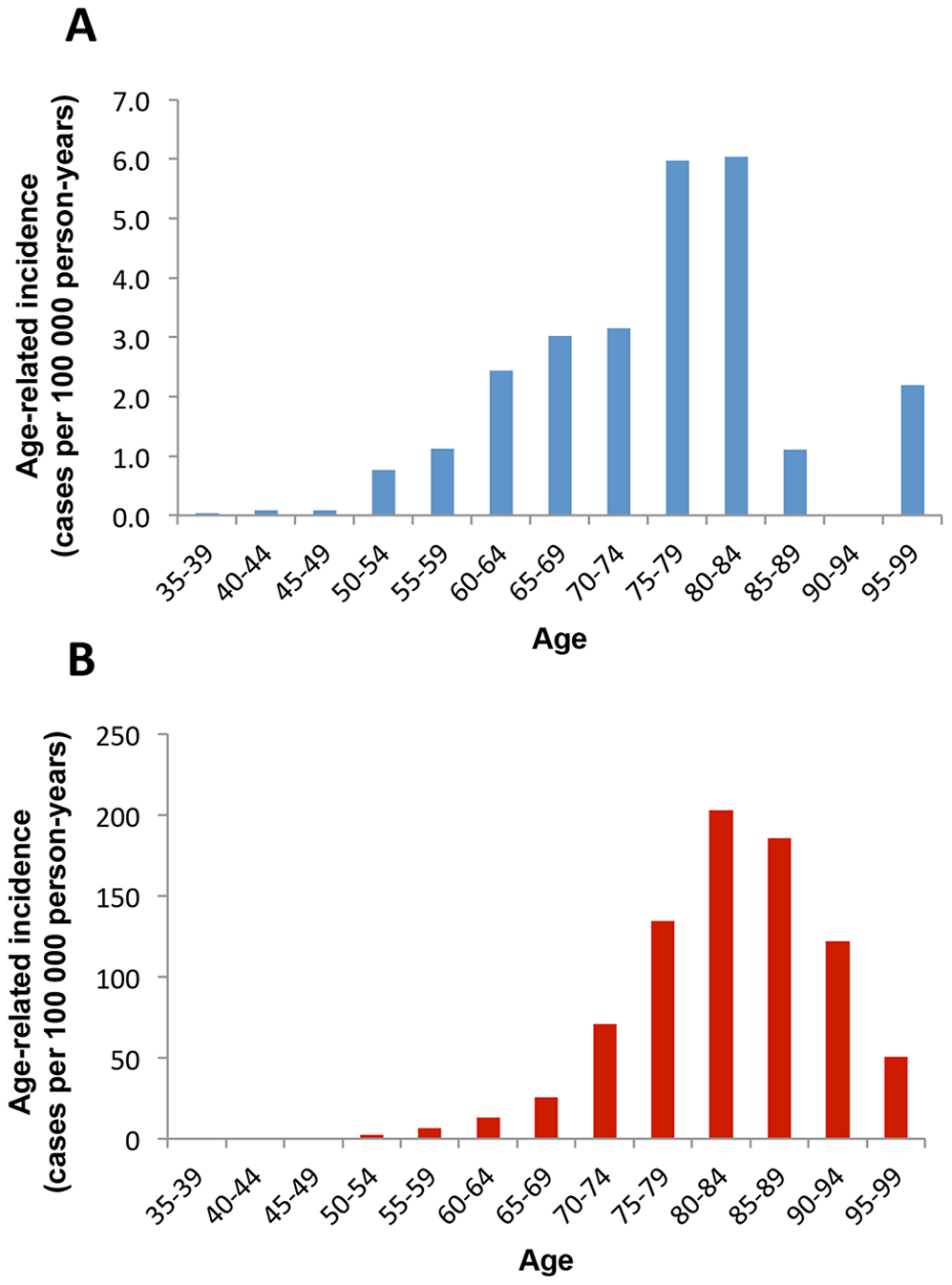
Age-related incidence in FTD and AD. The figures show the incidence of FTD (A; blue bars) and AD (B; red bars) in relation to age (years). Values for incidence are given as cases per 100 000 person-years in each 5 year age cohort.

Considering that the younger age cohorts are larger, the age-related incidence of FTD was calculated and compared to that of patients with AD (cases of mixed dementia were excluded). Both for FTD and AD, the highest incidence occurred in the age group 80–84 years at 6.04 and 202 cases per 100 000 person-years, respectively (Fig 2). However, the age distribution differed between the two disorders, with a higher proportion of early-onset cases in FTD, and the average age at diagnosis was significantly higher in AD (77.4 years) compared to FTD (69.6 years) (p<0.0001). For comparison with previous studies on incidence, the incidence of FTD in the age groups 30–64 (early onset) and 65–99 (late onset) years was also calculated. The incidence was 0.64 and 3.44 per 100 000 person-years in early-onset and late-onset FTD, respectively.

### Family history

**Figure 3:**
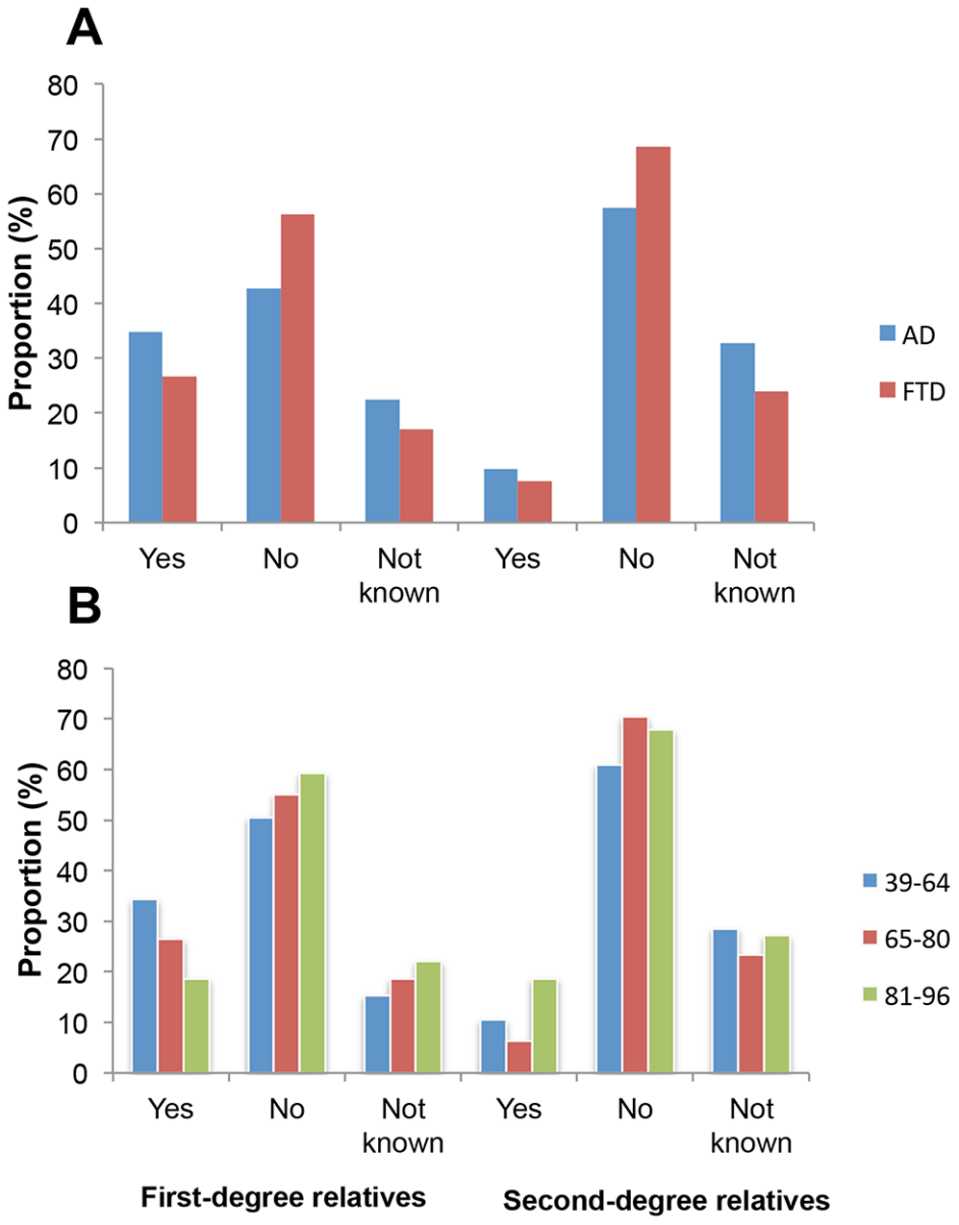
Family history in FTD and AD. The data shown in (A) represents the response to questions on the presence of dementia in first-degree and second-degree relatives of patients diagnosed with AD (blue bars) and FTD (red bars). In (B), the responses in FTD cases are divided into age cohorts: 39–64, 65–80, and 81–96 years. For classification purposes, the responses were divided into three categories: Positive family history (Yes), negative family history (No/None known) and Not known (reserved for when questions about family history have not been asked alternatively no response registered).

The number of FTD cases with a family history of dementia was recorded and compared to AD cases (Fig 3A). While 26.7% of FTD cases had a first-degree relative with dementia, only 7.6% had a second-degree relative and the number of FTD cases with both first- and second-degree relatives with dementia was even lower at 3.7%, similar to the frequencies of a positive family history for dementia in all patients diagnosed with dementia and registered in SveDem 2008–2011 (first-degree 29.4%, second-degree 7.6%). There was a significantly higher proportion of cases with a positive family history in AD than in FTD for both first-degree (34.8% vs 26.7%; p<0.0001) and second-degree relatives (9.8% vs 7.6%; p = 0.033) (Fig 3A). We also compared family history in different age groups for cases with FTD. Apart from a borderline-significant trend (p = 0.051) towards a higher proportion of cases with a positive family history in first-degree relatives of early onset cases (39–64 years), there were no larger differences between the different age groups (Fig 3B). Similarly, for AD, there was no major difference in family history between early and late-onset cases (data not shown).

## Discussion

In the largest study of age-related incidence to date, using a large community-based registry, we demonstrate that the incidence of clinically diagnosed FTD increases with age as has been previously reported in smaller populations [14], [16]. In a consecutive study of 100 patients with SD, 46% were diagnosed after the age of 65 years [27], which also indicates that late-onset cases of FTD complex disorders might be more common than previously thought. Although the average age at diagnosis was significantly lower for FTD than AD, both disorders showed a maximum age-related incidence in the same age cohort (80–84 years). This finding suggests that increasing age might be a major risk factor in FTD, as in other major neurodegenerative disorders such as AD and PD [28], [29]. It should be pointed out, however, that most previous studies have used age at onset rather than age at diagnosis which was used in this study. Although the use of age at onset avoids problems of patient and doctor delay, the estimation of time from onset to diagnosis is notoriously difficult and might be subject to very different interpretation from case to case. Only cases diagnosed with FTD in each respective year are registered in SveDem, thus avoiding the risk of inclusion of patients with a previous diagnosis of FTD.

While it is well documented that AD affects more women than men, and the reverse is true for DLB/PDD, there is conflicting data on the gender ratio in FTD [1], [6], [8], [9]. There was only a slight preponderance of women (56%) than men that received a diagnosis of FTD in the present study, supporting a more equal sex distribution in FTD than in AD, PDD or DLB. While data from SveDem reflects the distribution in the general population, previous discrepancies in gender ratio might reflect differences in recruitment strategy and the population studied.

The presence of a first-degree relative with dementia is common in both AD and FTD, suggesting that genetic risk factors are important in both these disorders [20], [30]. In FTD, several genetic mutations have been described that can lead to autosomal dominant hereditary disease [22]. Previous estimates of the proportion of familial FTD has varied between 10–40% of cases [1]–[2], [17]–[22], although the high community prevalence of other types of dementia in the elderly might make estimates of the proportion of familial cases uncertain [2].

The results of the present study confirm a high degree of positive family history in first-degree relatives of cases with AD and FTD. Cases with second-degree relatives were uncommon and autosomal dominant mutations have more recently been estimated to account for only around 10% of cases with AD and FTD [2], [21]. Data from SveDem also suggests that the proportion of cases with a positive family history is similar in FTD and AD.

The presence of a positive family history in FTD might, however, have been underestimated, especially in second-degree relatives. The questions used in SveDem do not specifically address the symptoms specific for FTD, and a significant proportion (15–25%) of responses were either “Not known” or data was missing. On the other hand, previous estimates of a high prevalence of positive family history in FTD might have been influenced by referral bias and research interest in familial cases as well as the high prevalence of dementia in the general population [2]. Another possible explanation for the lower proportion of familial FTD cases in SveDem could be that the available data does not differentiate between cases with bvFTD and the primary progressive aphasias (SD and PNFA). Autosomal dominant mutations and a positive family history are more common in bvFTD than in either SD or PNFA [22].

The main limitation of the present study is that the clinical data leading to diagnosis is not available. Consequently, it is not possible to control the validity of the diagnosis in each individual case. Furthermore, neuropathological confirmation of the diagnoses was not available. However, almost all diagnoses of FTD in the SveDem cohort were made at specialist centres. High clinicopathological concordance in early-onset dementia, with up to 97% specificity for bvFTD, has recently been demonstrated in a highly specialized centre [31]. To minimize inclusion of frontotemporal syndromes with ambiguous cause only cases with ICD-10 codes according to national consensus were used. More important, analysis of data from only those cases diagnosed in specialist centres and that included both cognitive testing and neuroimaging demonstrated identical age distribution compared to the whole FTD cohort. Neuroimaging greatly increases the specificity of a clinical diagnosis of FTD [32] and pathological findings on structural or functional imaging is a requirement for a diagnosis of probable FTD in the international consensus criteria published in 2011 [25]. Furthermore, lumbar puncture for analysis of AD biomarkers was performed in a high proportion of cases which increases detection of cases with underlying AD pathology [33].

The number of FTD diagnoses differed greatly between participating centres and interest and experience in FTD appears to be of greater importance for the diagnosis of FTD than the diagnostic procedures used. It is thus likely that FTD is still underdiagnosed in Sweden and this might be even more pronounced in the elderly. This is also supported by the relatively low incidence of FTD seen compared to previously published estimates [9], [14].

There are several possible reasons why FTD might be underdiagnosed in the elderly: First, the 1998 diagnostic criteria lists onset before 65 years of age as one of the supportive criteria and late-onset cases are stated as being rare [4], which might lead to bias against the diagnosis of FTD in elderly patients with behavioural symptoms. Second, the behavioural symptoms of FTD might be more disruptive and noticeable in occupational and family settings, thereby attracting more clinical attention in early-onset cases. Third, many memory clinics have a focus on early-onset cases which leads to referral bias. Fourth, as the incidence of AD increases very sharply with age and the ratio between cases of AD and FTD is much lower in early-onset dementia, there could be a greater recognition of FTD cases in younger age cohorts. Finally, there is accumulating evidence that the clinical and pathological features of FTD in the elderly differs from that of early-onset FTD, with memory problems and hippocampal sclerosis being more common, and frontal lobar atrophy less pronounced, in older patients [34], [35]. In support of this, the cases that failed to meet the new international consensus clinical criteria in a validation study were significantly older than the patients that fulfilled the criteria [25]. Taken together, symptoms of frontal lobe dysfunction in the elderly might often be attributed to other causes than FTD, such as VaD or AD. Prospective cohort studies, including neuropathological confirmation of the diagnosis, will be needed to confirm the findings in this study.

In summary, data from SveDem suggest that increasing age is an important risk factor in FTD, as for other neurodegenerative disorders. The increased recognition of FTD in the elderly has important consequences for dementia care. Compared to AD, patients with FTD often require other strategies for psychosocial support and nursing [2], have no effect of treatments with choline esterase inhibitors or memantin [2], [36], and are unsuitable as drivers at an earlier stage of the disease process compared to AD [37]. As the majority of patients with FTD in SveDem were above 65 years at diagnosis, our findings could also be important in the recruitment of patients for clinical trials.
